# What is the real number of Lyme disease cases in Canada?

**DOI:** 10.1186/s12889-019-7219-x

**Published:** 2019-06-28

**Authors:** N. H. Ogden, C. Bouchard, J. Badcock, M. A. Drebot, S. P. Elias, T. F. Hatchette, J. K. Koffi, P. A. Leighton, L. R. Lindsay, C. B. Lubelczyk, A. S. Peregrine, R. P. Smith, D. Webster

**Affiliations:** 1 Public Health Risk Sciences Division, National Microbiology Laboratory, Public Health Agency of Canada, St. Hyacinthe, Canada; 2 Office of the Chief Medical Officer of Health, New Brunswick Department of Health, Fredericton, Canada; 3 Zoonotic Diseases and Special Pathogens Division, National Microbiology Laboratory, Public Health Agency of Canada, Winnipeg, Canada; 4 Maine Medical Center Research Institute, Scarborough, ME USA; 5 Department of Pathology and Laboratory Medicine, Nova Scotia Health Authority and Dalhousie University, Halifax, NS Canada; 6 Policy Integration and Zoonoses Division, Centre for Food-Borne, Environmental and Zoonotic Diseases, Public Health Agency of Canada, Ottawa, Canada; 7 Département de pathologie et microbiologie, and Groupe de recherche en épidémiologie des zoonoses et santé publique (GREZOSP), Faculté de médecine vétérinaire, Université de Montréal, Québec, Canada; 8 Department of Pathobiology, Ontario Veterinary College, University of Guelph, Guelph, Canada; 9 Department of Medicine, Division of Infectious Diseases, Faculty of Medicine, Saint John Regional Hospital, Dalhousie University, Saint John, New Brunswick Canada

**Keywords:** Lyme disease, *Borrelia burgdorferi*, Surveillance, Under-reporting, Canada

## Abstract

**Background:**

Lyme disease is emerging in Canada due to expansion of the range of the tick vector *Ixodes scapularis* from the United States. National surveillance for human Lyme disease cases began in Canada in 2009. Reported numbers of cases increased from 144 cases in 2009 to 2025 in 2017. It has been claimed that few (< 10%) Lyme disease cases are reported associated with i) supposed under-diagnosis resulting from perceived inadequacies of serological testing for Lyme disease, ii) expectation that incidence in Canadian provinces and neighbouring US states should be similar, and iii) analysis of serological responses of dogs to the agent of Lyme disease, *Borrelia burgdorferi*.

We argue that performance of serological testing for Lyme disease is well studied, and variations in test performance at different disease stages are accounted for in clinical diagnosis of Lyme disease, and in surveillance case definitions. Extensive surveillance for tick vectors has taken place in Canada providing a clear picture of the emergence of risk in the Canadian environment. This surveillance shows that the geographic scope of *I. scapularis* populations and Lyme disease risk is limited but increasing in Canada. The reported incidence of Lyme disease in Canada is consistent with this pattern of environmental risk, and the differences in Lyme disease incidence between US states and neighbouring Canadian provinces are consistent with geographic differences in environmental risk. Data on serological responses in dogs from Canada and the US are consistent with known differences in environmental risk, and in numbers of reported Lyme disease cases, between the US and Canada.

**Conclusion:**

The high level of consistency in data from human case and tick surveillance, and data on serological responses in dogs, suggests that a high degree of under-reporting in Canada is unlikely. We speculate that approximately one third of cases are reported in regions of emergence of Lyme disease, although prospective studies are needed to fully quantify under-reporting. In the meantime, surveillance continues to identify and track the ongoing emergence of Lyme disease, and the risk to the public, in Canada.

## Background

Lyme disease (LD), caused by the bacterium *Borrelia burgdorferi* sensu stricto (for simplicity referred to hereafter as *Borrelia burgdorferi*), is an emerging infectious disease in eastern and central Canada due to the northward spread of the tick vector *Ixodes scapularis* [1] (also known as the blacklegged tick). Lyme disease is a zoonosis, and the ecology of this disease has been extensively reviewed (summarized in [2]). The natural reservoirs of *B. burgdorferi* are small and medium sized wild mammals (particularly mice of the genus *Peromyscus*) and birds. Immature *Ixodes* species ticks feed on these hosts and maintain *B. burgdorferi* transmission cycles. Deer are not reservoirs for *B. burgdorferi* but are the hosts for adult female ticks, so LD risk is indirectly related to deer densities. The vector ticks require woodland habitat because woodlands provide suitable densities of their wild animal hosts, and because they provide duff layer refuges that permit ticks to survive extremes of temperature in winter and summer during periods that they are not feeding on hosts. LD emerged in northeastern and upper Midwestern US in the 1970s, likely associated with changes in land use that resulted in increased woodlands allowing deer and then tick and *B. burgdorferi* populations to expand out of refugia [2]. Range expansion is facilitated by dispersal of ticks and bacterium over short distances by terrestrial hosts, and long distances by migratory birds [3]. The latter may have been particularly important in seeding *I. scapularis* and *B. burgdorferi* populations in Canada, as migratory birds leap-frog geographic barriers to terrestrial host movements such as the Great Lakes, the Appalachian Mountains and the sea [4–7]. Northward range expansion of *I. scapularis* into Canada, and subsequent emergence of LD, have been associated with effects of climate change on the biology of the tick; multiple studies have identified associations between temperature and the spatio-temporal pattern of spread of the ticks [8]. However, effects of climate warming on hosts of *I. scapularis* and *B. burgdorferi* may also be contributing to LD emergence [9]. In British Columbia, the vector of *B. burgdorferi* is *Ixodes pacificus* (the western blacklegged tick), which has been widespread in the south of the province for many years [10]. The risk of acquiring LD from bites of this tick species is considerably lower than from *I. scapularis* in the east, as few *I. pacificus* ticks are infected and they are less likely to bite humans than *I. scapularis* [11]. Since LD became nationally notifiable in Canada in 2009, the number of reported cases has increased more than tenfold from 144 cases to 2025 cases reported in 2017 [12]. This increase in incidence is consistent with the range spread of *I. scapularis* ticks in Canada [13], but other factors may modulate the risk of LD to the Canadian public via effects on rates of their exposure to tick bites. These include forest fragmentation, construction of residences in newly-established LD risk areas, and changes in knowledge and perception of LD risk and prevention methods [9, 14, 15]. It is unlikely that all LD cases are reported, however the precise rate of under reporting of LD in Canada is not known.

Infectious disease surveillance systems aim to elucidate patterns of a disease within a population and, by understanding these, inform public health policies and programs to reduce risk from that disease [16]. Surveillance systems often underestimate the “true” frequency of a disease in a population due to i) under-ascertainment (i.e. not all cases seek health care), and ii) under-reporting (i.e. not all symptomatic cases that have sought health care are captured by the surveillance system) [17]. Some definitions of under-ascertainment include cases that are not correctly diagnosed [17]. In some surveillance systems, the rate of under-reporting can be high (e.g. less than one 100th of infectious gastrointestinal illnesses were reported in Ontario in the 1990s [18]). However, if under-reporting is equal in the groups assessed by the surveillance system, trends should still be detectable and can be extrapolated to the population as a whole. If under-reporting is not equal, valid analyses are still possible if the inequalities are known and can be adjusted for, allowing estimation of the burden of illness [18, 19].

The under-reporting of LD cases through passive surveillance in the United States has been evaluated in a number of studies with estimated ranges of reporting of 10–90%. In regions with emerging infections, only one third of cases are likely identified in surveillance, due to issues common to emerging diseases, including limited knowledge of physicians about the disease, case definitions and the need to report [20]. In US states with high incidence, up to 90% of LD cases may go unreported secondary to “reporting fatigue” by clinicians [21]. However, even with high levels of under-reporting, trends in incidence are captured [21]. Recently it has been suggested there is a very high degree of under-reporting of LD cases in human case surveillance in Canada (less than 1 in 10 cases reported) based on the poor sensitivity of diagnostic tests to detect cases in Canada, and lack of reporting of cases detected [22]. In this article we evaluate the extensive surveillance data available for Canada to explore the extent to which underreporting may be occurring here. In so doing we aim to highlight that public health in Canada is active and informed in understanding the risk of LD to the public.

## Main text

### Serological diagnosis failure as a potential source of under-detection and under-reporting

Some patient advocacy groups suggest that the “two-tier” serological algorithm used for LD diagnosis; screening enzyme immunoassays (EIA) followed by confirmatory immunoblot testing (and test interpretation criteria) recommended by US Centers for Disease Control and Prevention (CDC) and supported by the Association of Medical Microbiology and Infectious Diseases (AMMI) Canada and the Public Health Agency of Canada (PHAC), is too insensitive and misses many cases [22]. This assertion is based on 1) the use of test performance data that do not consider the stage of infection [23], and 2) comparisons of test results that use CDC-recommended methods and test interpretations, with those conducted using alternative methods in private laboratories in the US [24].

Numerous studies have shown that the performance of serological tests depends on the stage of infection. A recent systematic review shows that the two-tier algorithm is insensitive in early localized LD but performs well in late LD, at which point sensitivity approaches 100% [25]. If sensitivity is averaged across all stages of LD, the sensitivity of the diagnostic approach appears worse than it actually is [23]. Issues of sensitivity in early LD are well known, and are taken into consideration in recommendations to clinicians about i) when to request serological testing, ii) how to interpret test results, iii) the occasional need for repeat testing, and iv) the empiric treatment of patients in the face of negative test results. The latter is the correct course of action in patients with early localized infection with erythema migrans rash (EM) [26, 27]. In North America, there is considerable strain diversity of *B. burgdorferi* sensu stricto and it is possible that variation amongst infecting strains results in variations in the capacity of infections to be detected by some sero-diagnosis kits [28]. However, if so, this variation would likely impact serodiagnosis in Canada and neighbouring states equally. Studies in Canada identified strains that had not, at the time of the studies, been detected in the US [29]. However, this was because of lack of effort to explore strains in states bordering Canada, which is a gap that was recently addressed [30].

False positive EIA and immunoblot results are known to occur because of cross reactivity with other bacterial and viral infections [31–33] and a positive test result has poor predictive value when patients do not have symptoms consistent with LD, and/or when they have a low likelihood of exposure to infected ticks [34, 35]. Hatchette et al. [36] assessed the level of background sero-positivity (using the two-tier algorithm) in the Nova Scotia population using 1855 residual sera from healthy persons undergoing blood tests as part of a regular health check-up. Of these samples, 215 were positive by a screening whole cell EIA test, none of which were positive on the confirmatory immunoblots. The sensitivity of the whole cell EIA test approaches 100% in late LD [25], so it is likely that no or very few samples negative by the whole cell EIA test would have tested positive in the two-tier algorithm. Therefore, this study indicates that there is a very low level of background seropositivity using the two-tier approach in the Nova Scotia population (< 0.2%). However, there was a high level of background positivity in the screening EIA, which is consistent with other studies [37, 38].

These data highlight two key points regarding serological diagnosis of Lyme disease. First, false-positivity rates of the two-tier algorithm, when using CDC-recommended test interpretation criteria, are low. In contrast, false-positivity rates (including false positive results in healthy controls) reach > 50% in some of the private US “specialist” laboratories that use alternative test interpretation criteria [24], making positive test results from these laboratories (using their own interpretation criteria) of limited diagnostic value. Second, false positive test results with the screening assays do not equate to “missed” LD cases, and they do not indicate under-detection of LD cases by serological diagnosis in Canada, as has been suggested [22]. They are simply false-positive, which is precisely why a two-tier diagnostic approach is used to maximise specificity.

### What does Canadian national and provincial human case surveillance suggest about under-reporting?

Although LD had been a reportable disease in many provinces since the late 1980s, national human case surveillance for this infection in Canada began in 2009 [39, 40]. It comprises the National Notifiable Disease Surveillance System (NNDSS), and the Lyme Disease Enhanced Surveillance (LDES) system. The LDES was initiated by the Public Health Agency of Canada (PHAC) in 2010 in collaboration with some provinces to obtain more detailed data on LD cases.

Reporting of cases in national surveillance is distinct from clinical diagnosis. Cases reported in surveillance must meet criteria of clinical manifestations, laboratory test results (when appropriate), and (for confirmed cases) a history of possible exposure to infective tick bites. These criteria are necessarily more restrictive than those used for clinical diagnosis, and have been designed to minimise the numbers of cases reported that are not LD, and ensure valid and reliable trends in LD incidence that require public health action [39]. The high likelihood of negative serological test results in early localized infection does not mean that patients are not diagnosed and treated by their clinicians, or that these cases are not reported. Cases presenting with an EM rash and without laboratory evidence of infection can be reported as this is one of the alternative national surveillance case definition criteria [39, 40]. The rates at which under- and over-diagnosis of LD may be occurring due to limitations of front-line physician awareness of clinical and laboratory diagnosis recommendations [41, 42] is another matter, and will be best estimated by well-designed prospective studies.

Amongst the objectives of the LDES system is the collection of data on the clinical manifestations of reported cases. These data are used to identify the proportion of cases that are early LD (i.e. EM rash), early disseminated LD (multiple EM, neuroborreliosis, or Lyme carditis), or late disseminated LD (Lyme arthritis) [13, 40]. Analysis of these surveillance data shows that a low proportion are reported in the early LD stage (approximately 14% of cases acquired in known LD risk areas). However, in the US 70% of cases are reported at this stage [40]. A similar analysis of surveillance data from Ontario produced the same result [43] and together these suggest that in Canada either EM cases are being detected and diagnosed but not reported, or that LD is frequently only diagnosed in the disseminated LD stages. In data from the LDES system, previous manifestations of EM were recorded for many cases of disseminated LD and overall the proportion of cases with a history of EM was the expected 70% [40], suggesting that much of the shortfall is due to LD not being diagnosed at the EM stage. This is consistent with studies on physician awareness [41], and with the observation that over time, the proportion of reported cases that are early LD has increased as efforts to improve physician and public awareness have been intensified. It also provides evidence of the utility of the surveillance system in identifying policies needed (in this case physician awareness [44]) to better protect Canadians from emerging LD.

### Why is reported incidence in Canada much lower than in neighbouring US states?

LD emerged in the southern parts of New England states in the 1970s [45]. Re-forestation of farmland in the twentieth century allowed the expansion of the geographic range and abundance of *I. scapularis* and *B. burgdorferi* populations out of refugia in the northeast and upper Midwest of the US resulting in increased transmission of the infection to humans [2]. This range expansion has now reached southern Canada, facilitated by dispersion of *I. scapularis* and *B. burgdorferi* by migratory birds from the US [6], and a warming climate to increase environmental suitability for *I. scapularis* at the northern edge of the tick’s range [8]. This has resulted in the emergence of LD on the Canadian side of the border. However, it is very clear that the risk of acquiring LD from the environment (i.e. the risk of acquiring bites of infected ticks) is different in Canada than in neighbouring US states. This difference is due to: i) different stages of emergence and the extent of geographic spread of *I. scapularis* and *B. burgdorferi* (both currently limited in distribution and less abundant/prevalent in Canada compared to southern parts of neighbouring US states), and ii) high levels of exposure of the US population in the southern parts of these states where both population densities and LD risk are higher. In addition, some differences in case definitions and reporting must be considered when comparing LD incidence in the two surveillance systems.

We have a good understanding of the level of environmental risk for LD due to extensive passive and active field surveillance for ticks in the US and Canada. A country-wide assessment of LD risk in the US environment was conducted in 2004 [46] with visits to 95 sites in 37 states east of the 100th meridian, and this revealed the greatest risk in states of the upper Midwest and northeast. Many of these states border Canada and many, including Maine, Minnesota, New Hampshire, New York, Pennsylvania, Vermont, and Wisconsin, are now considered by CDC as “high incidence” states (those with an incidence of confirmed LD cases of > 10/100,000 [47]). However, the risk within those states is not homogeneous. Multiple studies since the 1990s, in states bordering Canada, have collectively identified concentration of ticks and LD risk in the southern parts of the states that are distant from the border with Canada. This risk is changing, and ongoing northward spread of ticks and LD risk, facilitated by movements of hosts of ticks and *B. burgdorferi*, from the southern parts of the states has been identified in multiple studies (Michigan: [48, 49]; Minnesota: [50]; Wisconsin: [51]; New York State: [3, 52]; Vermont: [53]; New Hampshire: [54]; Maine: [55–57]; summarised in [58]).

**Fig. 1:**
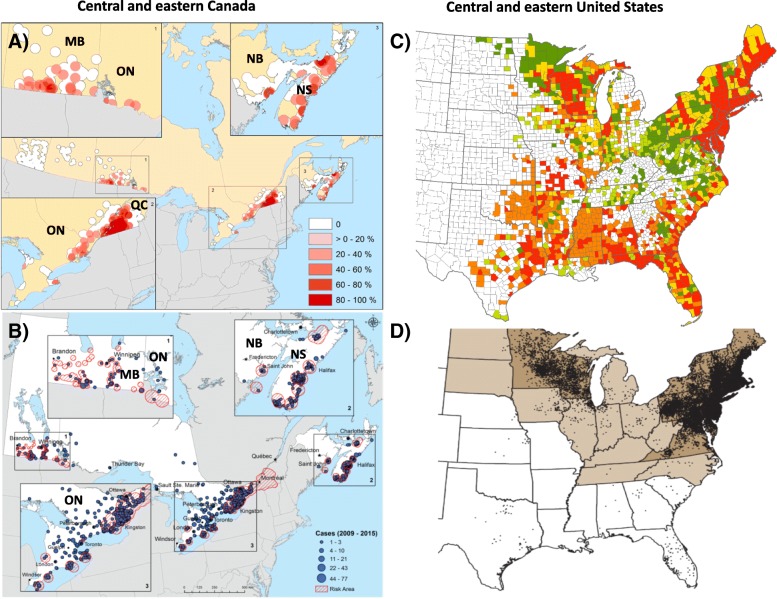
Surveillance for *I. scapularis* and associated human LD cases in Canada and the US. Occurrence of blacklegged tick populations detected in active field surveillance in central and eastern Canada from 2003 to 2012 are shown in panel **a**, reproduced with permission from [73]: the scale indicates the percent of sites positive for *I. scapularis*. The occurrence of human LD cases identified in enhanced surveillance in Canada from 2009 to 2012 are shown in panel **b** (reproduced unchanged from [13]), noting that locations of acquisition of cases in the province of Quebec are not available. MB = Manitoba, ON = Ontario, QC = Quebec, NB = New Brunswick, NS = Nova Scotia. Occurrence of US counties with *I. scapularis* in a summary of tick surveillance data from the US (using the data in [58]) is shown in panel **c**. This map shows the evolution of *I. scapularis* populations in the US from data compiled in 1999 and in 2016. Red and orange fill indicates counties that were considered, respectively, as having “established” or “reported” *I. scapularis* in 1999 (see [58] for the definitions). Green and yellow fill respectively indicate counties that changed from no records to established or from reported to established by 2016. The occurrence of LD cases reported in national surveillance in the US (by county of residence) is shown in panel **d** (reproduced from [47])

During the 1980s, the only known location for *I. scapularis* populations in Canada was at Long Point, Ontario on the north shore of Lake Erie [59]. With reports of additional *I. scapularis* populations on the shores of Lake Erie [60, 61], more systematic active field surveillance was initiated. Currently, southeastern and south central Canada are regions affected by the range expansion of *I. scapularis* and *B. burgdorferi* (Fig. 1). The status of LD risk in the Canadian environment, and the resulting incidence of LD, have been studied over the last three decades with an intensity that is possibly unique for a tick-borne disease of public health importance. Two complementary methods of tick surveillance have been employed: passive tick surveillance, and active field surveillance for ticks.

Passive tick surveillance in Canada is a Federal-Provincial collaboration that involves voluntary submission of ticks found on domesticated animals and humans by participating veterinary and medical clinics and in some jurisdictions from the general public [62]. It began in 1990, has mostly national coverage, and has provided a uniquely long-term database to assess the emergence of tick populations in Canada [63]. Active field surveillance involves the collection of ticks by drag/flag sampling and capture and examination of animal hosts for ticks and is the “gold standard” method of identifying the entomological risk of LD (i.e. the presence of established tick populations and cycles of *B. burgdorferi* transmission) [64]. We estimate that > 2500 individual field sites (more than double the number of meteorological stations in the whole of Canada [44]) have been visited for active field surveillance in southern Canada east of the Rockies since 2008 to follow the evolution of tick populations and infection prevalence in ticks. Many of these sites are regularly revisited, and new sites are added each year.

Active field surveillance for ticks, passive tick surveillance and human case surveillance are integrated. Information on tick abundance from passive surveillance often acts as a trigger for active field surveillance, as do clusters of human cases in locations where ticks have not been previously found. Together the results of these three types of surveillance have been regularly synthesised in peer-reviewed publications, and on provincial and federal websites, to provide the public with knowledge of where risk from LD occurs in Canada (Table 1).

**Table 1:** Examples of studies on, and syntheses of, surveillance for *I. scapularis* ticks, *B. burgdorferi* and Lyme disease cases in Canada. These include studies conducted or funded by Federal or Provincial public health organisations. These do not include articles on detection of ticks and pathogens by researchers independent of public health organisations. *A* active field surveillance for ticks, *P* passive tick surveillance, *H* human case surveillance, *Ph* phylogenetic analysis, *S* synthesis. Province abbreviations are *AB* Alberta, *BC* British Columbia, *SK* Saskatchewan, *MB* Manitoba, *ON* Ontario, *QC* Quebec, *NB* New Brunswick, *NS* Nova Scotia

Year of surveillance/analysis	Province	Type of surveillance data	Article/website and reference number
1990–2003	Canada	P	Ogden et al. 2006 Ref [62]
1990–2008	Canada	A, P	Leighton et al. 2012 Ref [63]
1991–2012	Canada	A, P	Bouchard et al. 2015 Ref [81]
1996–2008	QC	A, P, Ph	Ogden et al. 2010 Ref [67]
1996–2010	Canada	A, P, Ph	Ogden et al. 2013 Ref [68]
2004–2013	Canada	A, P	Ogden et al. 2014 Ref [64]
2005–2014	ON	H	Johnson et al. 2018 Ref [43]
2005–2015	AB, SK, MB	A	Gabriele-Rivet et al. 2017 Ref [82]
2007	QC	A	Ogden et al. 2008 Ref [83]
2007–2008	QC	A	Bouchard et al. 2011 Ref [84]
2007–2008	QC	A, P	Koffi et al. 2012 Ref [85]
2007–2008	QC	A, P	Bouchard et al. 2013 Ref [86]
2007–2012	QC	A	Bouchard et al. 2018 Ref [87]
2008–2012	ON	P	Nelder et al. 2017 Ref [88]
2008–2014	QC	P	Gasmi et al. 2016 Ref [89]
2009–2010	ON	A	Werden et al. 2014 Ref [90]
2009–2012	Canada	A, H	Ogden et al. 2015 Ref [40]
2009–2014	QC	A, P, H	Ripoche et al. 2018 Ref [91]
2009–2015	Canada	A, H	Gasmi et al. 2017 Ref [13]
2010–2016	ON	P, H	Kulkarni et al. 2019 Ref [92]
2013	QC	A	Ripoche et al. 2018 Ref [93]
2013–2017	ON	A, P	Schillberg et al. 2018 Ref [94]
2013–2014	BC	A	Morshed et al. 2015 Ref [95]
2013–2017	ON	A, P	Soucy et al. 2018 Ref [96]
2014	NB	A	Gabriele-Rivet et al. 2015 Ref [66]
2014	ON	A	Clow et al. 2016 Ref [97]
2014–2016	ON	A	Clow et al. 2017 Ref [65]
To the present	AB	A, P	Alberta Health Ref [98]
To the present	BC	S	British Columbia Centre for Disease Control Ref [99]
To the present	SK	A, P, H	Saskatchewan Health Ref [100]
To the present	QC	A, P, H, S	INSPQ Ref [101]
To the present	ON	A	Public Health Ontario Ref [102]
To the present	MB	A, P, H, S	Manitoba Health, Seniors and Active Living Ref [103]
To the present	NS	A, P, H, S	Nova Scotia Department of Health and Wellness Ref [104]
To the present	NB	A, P	New Brunswick Health Ref [105]

The tick surveillance data provide a clear picture of the expansion of *I. scapularis* populations over the last two decades as an extension of the expanding LD risk in the US (Fig. 1). Expansion of the range of *I. scapularis* populations has been tracked year-on-year in both active and passive surveillance data [63, 65]. Currently, areas of Canada where *I. scapularis* populations have become established are increasing, but they are still somewhat limited (Fig. 1). The patterns of tick spread are geographically variable, with expansion occurring in a geographically uniform pattern in southeastern Ontario and the contiguous southern Quebec, and in southern Manitoba and the contiguous northwestern Ontario. In other areas *I. scapularis* populations have a more patchy occurrence, which may be due to limitations of the woodland habitats and densities of tick hosts in these regions [64, 66]. There are also three other elements of the emergence process which continue to increase the risk of acquiring LD behind the advancing front of range expansion. These are elements of the “maturity” of the establishment of ticks and *B. burgdorferi* transmission cycles: i) “infilling”, i.e. increasing proportions of woodland sites become occupied by ticks [65], ii) increased tick densities as the ticks continue to reproduce within the constraints of host abundance, and iii) increasing prevalence of infection in ticks [67, 68].

**Fig. 2:**
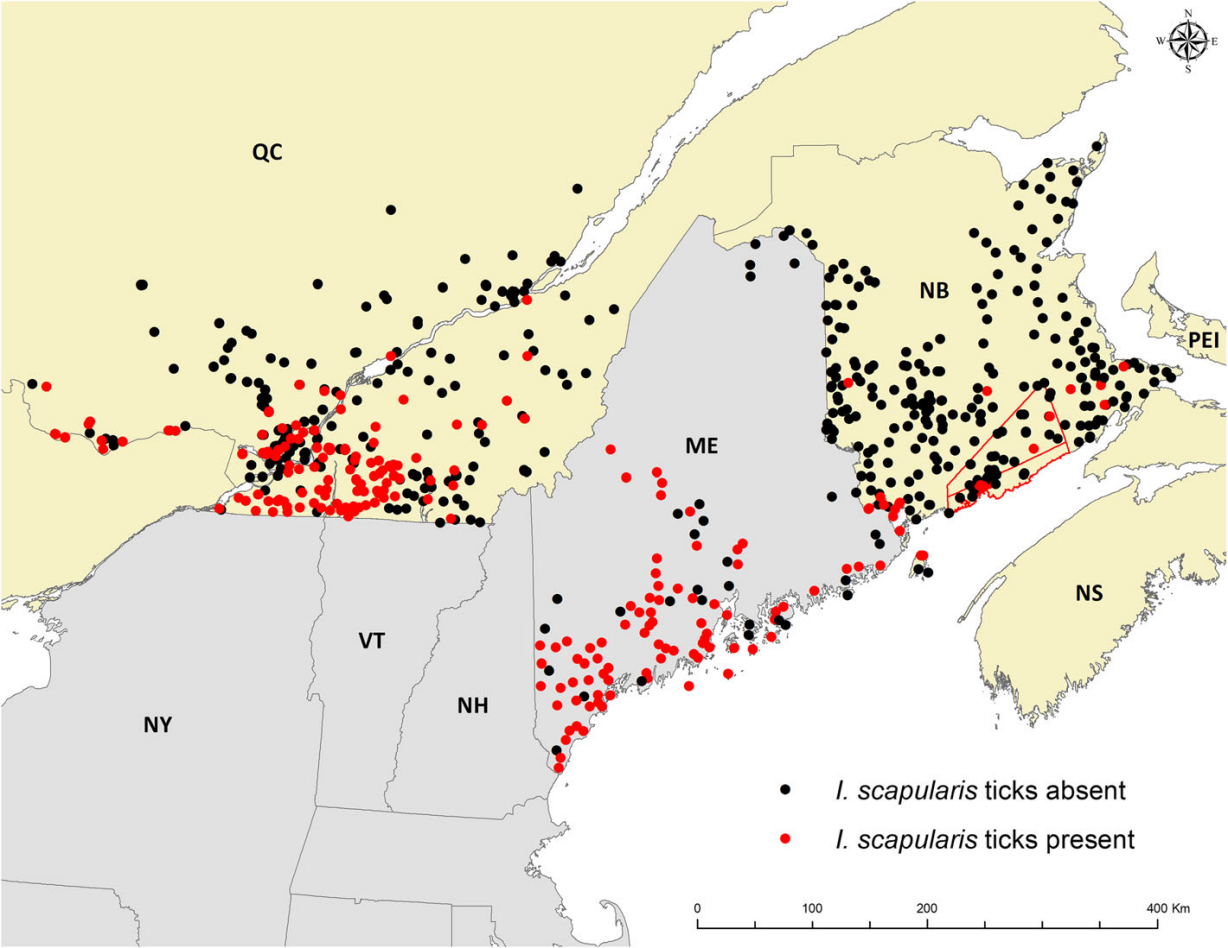
Sites of active field surveillance for ticks in the contiguous region of Maine, New Brunswick and Quebec. The status of known Lyme disease risk areas in 2017 in the neighbouring regions of Quebec (data from 2007 to 2017), New Brunswick (data from 2008 to 2017) and Maine (data from 1989 to 2017) is identified using similar drag sampling methodologies in active field surveillance for ticks. Red points indicate locations where *I. scapularis* populations have been found and black points indicate where surveillance has occurred but the ticks were not found. Data on surveillance in Quebec are presented with the permission of Institut national de santé publique du Québec and Ministère de la Santé et des Services sociaux, and New Brunswick data are presented with the permission of New Brunswick Department of Health. The red lines indicate the adjacent counties of Kings and St. John (to the south) in New Brunswick from where the majority of human LD cases are reported, and where canine seroprevalence was particularly high in 2015

Taken as a whole, the risk of acquiring LD, and the incidence of LD, is much higher in US states bordering Canada than in Canadian provinces. However, in the border regions the distributions of tick populations and risk locations for acquiring LD are similarly heterogeneous in both countries. A prime example is Maine, where in one of two counties bordering New Brunswick (Washington County) the incidence of LD in 2015 was 57.8 cases per 100,000 population. However, in Aroostook County, which lies just north of Washington County, and also borders New Brunswick, the incidence in 2015 was 2.8/100,000. This is lower than the incidence in New Brunswick in the same year; there were 1.7/100,000 cases reported in New Brunswick in 2015, which could equate with 3.4/100,000 if early LD cases had been reported in New Brunswick as they were in Maine (where approximately 50% of reported cases were early LD [69]). Differences in human case incidence reported in Maine, Quebec and New Brunswick [13] are clearly reflected in the heterogeneity of environmental risk for LD. Extensive active field surveillance for ticks and LD risk identifies presence in southern Quebec, and southern/coastal Maine, but absence from much of New Brunswick (Fig. 2).

Limited geographic scope of areas of LD risk in Canada, as well as “immaturity” of LD risk in terms of the proportion of woodlands affected, the density of ticks and the prevalence of infection in those ticks, is consistent with low but increasing incidence of reported human cases. The wide geographic scope and maturity of LD risk in the southern parts of the neighbouring US states results in expected differences in reported state-wide and province-wide LD incidence when comparisons are made across the border (Table 2). However, there is also expected similar incidence in some locations. Reproducing populations of vector ticks are not known to be established in either Alberta or Montana, and the low and similar incidence of reported cases is because the cases are travel acquired. The tick vector *I. pacificus* is widely established in both British Columbia and Washington state, but it is a vector that poses a low risk of LD for humans [11] and reported LD incidence is equally low on both sides of the border (Table 2).

**Table 2:** Comparison of reported human LD case incidence (per 100,000 population) in Canadian surveillance (probable and confirmed cases combined) in 2014 to 2016 [12, 13] with comparable data from the neighbouring US states (incidence for 2014 to 2015 obtained from [47] and for 2016 from https://www.cdc.gov/lyme/stats/tables.html, with State population estimates obtained from the US census: https://www.census.gov/data/tables/time-series/demo/popest/2010s-national-total.html#par_textimage_2011805803). These data were chosen as they are contemporaneous with similar data presented in [22]. Province abbreviations are *AB* Alberta, *BC* British Columbia, *MB* Manitoba, *ON* Ontario, *QC* Quebec, *NB* New Brunswick, *NS* Nova Scotia. State abbreviations are *MT* Montana, *ND* North Dakota, *MN* Minnesota, *WI* Wisconsin, *MI* Michigan, *NY* New York, *VT* Vermont, *NH* New Hampshire, *ME* Maine, *WA* Washington

Canada	Incidence in Canada			Neighbouring US States	Incidence in the US (fold difference)^a^		
	2014	2015	2016		2014	2015	2016
AB^b^	0.2	0.3	0.2	MT	0.5 (2.5)	0.2 (0.7)	1.2 (6.0)
MB	2.7	2.3	3.9	ND,MN	14.4 (5.3)	19.1 (8.3)	20.9 (5.4)
ON	1.7	2.7	2.7	MN,WI,MI,NY	11.8 (6.9)	14.3 (5.3)	13.6 (5.0)
QC	1.5	1.9	2.1	NY,VT,NH,ME	23.3 (15.5)	21.7 (11.4)	22.8 (10.9)
NB	0.7	1.5	1.5	ME	87.9 (62.8^c^)	74.7 (24.9^c^)	86.4 (28.8^c^)
NS	12.1	26.1	34.4	ME	87.9 (7.3)	74.7 (2.9)	86.4 (2.5)
BC	0.1	0.3	0.8	WA	0.1 (1.0)	0.2 (0.7)	0.2 (0.3)

^a^The difference between incidence in the US States compared to Canadian Provinces. Note that there are slight differences amongst Canadian provinces in the data provided in surveillance, and consequently the capacity to separate endemically acquired from travel-acquired cases. ^b^Only travel-related cases have been found in Alberta and neighbouring US states. ^c^This difference accounts for approximately 50% of reported cases in Maine being EM rash only without laboratory test results (https://www.maine.gov/dhhs/mecdc/infectious-disease/epi/vector-borne/lyme/documents/2017-Lyme-Surveillance-Report.pdf), which would not at the time have been reported in NB

### Does prevalence of seropositivity in dogs provide evidence for under-reporting of LD in Canada?

It has been common practice across Canada and the US for veterinarians to test dogs for seroconversion to vector-borne infections (heartworm, LD, ehrlichiosis and anaplasmosis) with a pet-side “SNAP” test when they present for routine check ups (the latest iteration being the IDEXX company’s SNAP 4Dx Plus test: [70, 71]). The LD component of the test is equivalent to the C6 Enzyme-Linked Immunosorbent Assay (ELISA), which has high specificity and sensitivity in detecting antibody responses to *B. burgdorferi* in dogs [72]. Consequently, routine testing across a large geographic area of North America over a number of years has produced data of interest for understanding LD risk. A number of studies in the US have found correlation between the prevalence of positive test results in dogs and the reported incidence of LD in humans from the same geographic regions over the same time periods. Analysis of similar data may be a useful adjunct to understanding LD risk in Canada, although results from previous studies, and data collected prospectively, need to be analysed carefully. Seroprevalence in dogs is a measure of the level of entomological risk (i.e. tick-borne infection) to which the dog population has been exposed over a number of years, as dogs (like humans) likely remain seropositive for multiple years following infection [57]. Furthermore, while the C6 ELISA has high specificity (96.2%: [72]), up to 3.8% of positive test results may be false. Consequently, interpretation of low prevalence results in the tested population in Canada, which would have low pretest probability for infection (by being healthy dogs undergoing check-ups, and often being from regions where the likelihood of infection is low), need to be made with particular care. In addition, the possibility that dogs acquired infection during travel is not accounted for in the analyses as this information is not generally kept [72] although approximately 10% of positive test results may be associated with travel outside Canada [73].

Lloyd and Hawkins [22] used an estimate of 6% seropositivity of dogs in New Brunswick to directly estimate 2569 and 6475 human LD cases in 2014 (incidences of 344 and 867/100,000) using two different methods. They also used a complicated back calculation from seropositivity in dogs, through data from passive tick surveillance, to estimate 291 human LD cases in 2014 (an incidence of 39/100,000). These estimates do not match the detailed knowledge we have of LD risk in the environment in New Brunswick at that time. Mead et al. [74] used a ballpark canine seroprevalence cut-off of 5% for states with high and low human LD case incidence. However, in most studies, seroprevalence values in dogs lower than 10% are associated with low reported human case incidence. In studies conducted in Maine in 2007 [57], values for canine seroprevalence of less than 8% (3.5–7.9%) were recorded in the 6 northern counties, and greater than 8% (8.1–34.4%) in the remaining 10 southern counties. The mean human LD incidence in those counties with seroprevalence less than 8% was 2.7/100,000 (range 0–5.7), and 49.2/100,000 (range 10.3–87.2) for the southern counties where canine seroprevalence was greater than 8% (obtained using LD case data from CDC [75], and population data from the US census [76]). In “highly endemic” US states (human case incidence ranging from 10.8 to 69.1 reported cases/100,000 [47]), the prevalence of C6-positive dogs has been found to range from 7.1 to 19.8% [74, 77]. An earlier study showed that in towns in Massachusetts in which the canine seroprevalence was less than 10%, the mean incidence of LD was 3.2/100,000, although this study predated the use of the C6 ELISA [78]. This study, supported by more recent findings [79], showed that the relationship between canine seroprevalence and human LD incidence is non-linear, with human LD incidence falling below 2/100,000 when canine seroprevalence was less than 6%. Three Canadian studies on canine seroprevalence obtained using the IDEXX SNAP test have been published; Villeneuve et al. [73] examining IDEXX data from 2008, Herrin et al. [72] examining similar data from 2013 to 2014, and Evason et al. [80] examining the data from 2008 to 2015. In 2008, the seroprevalence in Canadian dogs was 0.72%, and in 2013–2014 the combined seroprevalence was 2.5% with increases in seroprevalence in the Maritimes, Quebec, Ontario and Manitoba from 2008 to 2013–2014 (Table 3). The low but increasing seroprevalence in dogs is consistent with our knowledge of the level of entomological risk of LD in Canada obtained by tick surveillance. One outlier datum presented in Evason et al. [80] is a seroprevalence value of 8.8% (53/604) in dogs from New Brunswick in 2015, which is more than double the seroprevalence in dogs from this province in 2013 to 2014 combined (Table 3). Closer inspection of the data provided to us by the authors show, however, that many of the positive test results came from veterinary practices in the neighbouring New Brunswick counties of St John and Kings, which are identified by active field surveillance for ticks as LD risk areas (Fig. 2). The prevalence in these samples is clearly much higher (35/207, 16.9%), than in those from the rest of the province where seroprevalence remains at a level comparable to that in 2013–2014 (18/397, 4.5%). This is consistent with the majority of human LD cases (13 of 20 for the whole province) being reported as having been acquired in these counties from 2013 to 2015, with a maximum incidence in 2015 of 5.6/100,000, versus 2.0/100,000 for the rest of the province (personal communication, J Badcock). Human LD incidence continues to rise in this region of New Brunswick and by 2017 the incidence in these counties (combined) had risen to 14.7/100,000 (personal communication, J Badcock). Consistent with [74], these data suggest that canine seroprevalence values may be able to identify emerging hotspots of LD risk. However, they also indicate that careful analysis of the spatial pattern of the data is required to interpret seroprevalence values across broad geographic areas, particularly when the number of samples is relatively small. Overall, the reported LD incidence in humans in Canada is consistent with the observed low prevalence of positive canine sera, which does not provide any evidence for the presence of significant under-reporting of human LD cases.

**Table 3:** Seroprevalence of *B. burgdorferi* antibodies in dogs from Canada using the IDEXX Snap test in 2008 [73] and 2013–2014 [72], compared to human incidence of reported LD cases in Canadian provinces (mean for 2013–2014 [13]). 95% CI = 95% confidence intervals. Province abbreviations are *BC* British Columbia, *AB* Alberta, *SK* Saskatchewan, *MB* Manitoba, *ON* Ontario, *QC* Quebec, *NB* New Brunswick, *NS* Nova Scotia, *PEI* Prince Edward Island

Province	2008 canine seroprevalence data (% and 95 CI)	2013–2014 canine seroprevalence data (% and 95 CI) [65]	Mean incidence (2013–2014) of human Lyme disease in Canadian provinces
BC	0 (0–0.09)		0.10
AB	0.17 (< 0.01–0.64)		0.35
SK	0.34 (< 0.01–1.24)	0.54 (0.01–3.30)	0.05
MB	1.90 (1.70–2.15)	2.4 (2.1–2.7)	2.5
ON	0.47 (0.42–0.53)	2.3 (2.2–2.4)	2.0
QC	0.57 (0.45–0.71)	2.8 (2.6–3.0)	1.6
NB	0.66 (0.01–3.63)	3.7 (2.9–4.7)	0.7
NS	2.15 (1.21–3.52)	15.7 (11.4–21.3)	14.1
PEI	10.0 (2.11–26.52)		0
Total for Canada	0.72 (0.67–0.78)	2.46 (2.37–2.55)	1.7

## Conclusion

Our comparisons of human case LD incidence, as reported in surveillance and as suggested by canine seroprevalence, in the US and Canada do not support the idea that there is a high degree of under-reporting (< 10% of cases reported) in Canadian human case surveillance. Even if it could be argued that these comparisons are themselves plagued by under-reporting, the extensive surveillance efforts conducted in Canada mean that we have a strong understanding of where LD risk is occurring, how it has evolved and in what ways it is similar to, and different from, that occurring in neighbouring US states. It is clear that, as for other reportable disease surveillance programs, under-reporting of LD occurs in Canada, and in part the surveillance is designed to discover this. However, tick surveillance data, combined with evidence from serological studies in dogs, do not suggest high levels of under-reporting in Canada. We speculate that the rate of under-reporting in Canada may be similar to that seen in regions where LD is emerging in the US [20]. The precise degree of under-reporting is unknown, and further prospective studies are needed to measure this.

## Data Availability

Tick surveillance data that support the findings of this study are available from Anne Kimpton and Dr. Jacqueline Badcock (of Institut national de santé publique du Québec and New Brunswick Department of Health respectively), and Dr. Robert Smith (Maine Medical Center Research Institute) but restrictions apply to the availability of these data, which were used under license for the current study, and so are not publicly available. These data are however available from the authors upon reasonable request and with permission of Institut national de santé publique du Québec, Ministère de la Santé et des Services sociaux, New Brunswick Department of Health and Maine Medical Center Research Institute. Data on distributions of *I. scapularis* in the US in Fig. 1 panel C are available from Dr. Rebecca Eisen, US CDC. Canine seroprevalence data for New Brunswick in 2015 are available from IDEXX Laboratories, Inc. (IDEXX Drive, Westbrook, Maine 04092, US), but restrictions apply to the availability of these data, which were used under license for the current study, and so are not publicly available. These data are however available from IDEXX Laboratories, Inc. upon reasonable request and with permission.

## Abbreviations

CDC: United States Centres for Disease Control & Prevention
EIA: Enzyme immune assay
ELISA: Enzyme linked immunosorbent assay
EM: Erythema migrans
INSPQ: Institut national de santé publique du Québec
LD: Lyme disease
PHAC: Public Health Agency of Canada
